# Synchrony analysis: application in early diagnosis, staging and prognosis of multiple sclerosis

**DOI:** 10.3389/fncom.2014.00073

**Published:** 2014-07-21

**Authors:** Zahra Ghanbari, Shahriar Gharibzadeh

**Affiliations:** Department of Biomedical Engineering, Amirkabir University of TechnologyTehran, Iran

**Keywords:** multiple sclerosis (MS), synchrony, early diagnosis, staging, prognosis, prediction

Multiple Sclerosis (MS) is an autoimmune disease caused by degeneration of the myelin sheath of large diameter fibers in the central nervous system. This will cause deficits in the conducting properties of nerves and also affect electrical signaling. As a result, in MS patients, nerve conduction will be slower than normal (Kandel et al., 2000).

Neural synchrony has been of great interest in neuroscience recently. In signal processing, synchrony refers to quantifying similarity, coherence or correlation among signals and could be measured using a variety of methods (Dauwels et al., 2010). Neural synchrony represents how synchronous the neurons are firing (Vialatte et al., 2008). It is proven that synchrony is an important feature of brain signals. Many neurological diseases are accompanied by abnormalities in neural synchrony (Dauwels et al., 2008).

For example, loss of synchrony among brain signals has been observed in disorders such as Parkinson's and Alzheimer's disease (AD) and was used for the purpose of diagnosis. On the other hand, increasing synchrony has been reported in disorders such as epileptic seizures (Vialatte et al., 2009).

Since perturbation in electrical signaling and slowing of nerve conduction are common among MS and the aforementioned diseases, it brings up the idea of using synchrony for MS as well. In addition, previous works on MS have reported loss of connectivity and synchronous function among different parts of patients' brains. It should be mentioned that most of the previous works were concentrated on the cognitive impairments caused by the disease, and they applied their methods on MEG signals (Arrondo et al., 2009; Hardmeier et al., 2012).

The other point which should be noted is that although MRI and ERP are both common tools in MS diagnosis and follow up, definite diagnosis cannot be made based on these criteria individually. In addition, MRI needs to be repeated (Greenberg et al., 2009; Longo et al., 2012) and it is not affordable and available in many situations. So, we should try to find a reliable solution.

According to the aforementioned points, we believe that recording electrical brain signals (particularly EEG and ERP) and calculating local and global synchrony among their channels may provide us with an individual tool for diagnosing MS. Actually, the idea we put forward is using calculated synchrony indices for the purpose of detection, classification and prediction on electrical brain signals. Of course, the previous results which investigated connectivity and synchronous function of brain parts support our idea (Arrondo et al., 2009; Hardmeier et al., 2012).

The proposed idea may also help us to detect MS in early stages. Additionally, we believe as impairments will increase by progression of the disease, synchrony measures may have significant differences in different stages of the disease. So, they could be useful for staging of the disease as well.

We also propose measuring synchrony among brain signals in the onset periods. It seems that there should be a correlation between the changes in synchrony measures and disease prognosis. In better words, based on the calculated synchrony indices, we can predict the trend of the disease. This would provide us with a clearer perspective of the possible efficiency of different management modalities (including medical and surgical). Additionally, based on the potential level of neural dyssynchrony the proposed idea can be useful in order to assess the efficiency of the selected treatments for both the patient and the physician. Surely experimental evaluations are needed to validate our hypothesis.

## Conflict of interest statement

The authors declare that the research was conducted in the absence of any commercial or financial relationships that could be construed as a potential conflict of interest.
